# Moralized language predicts hate speech on social media

**DOI:** 10.1093/pnasnexus/pgac281

**Published:** 2022-12-07

**Authors:** Kirill Solovev, Nicolas Pröllochs

**Affiliations:** JLU Giessen, Licher Straße 62, D-35394 Giessen, Germany; JLU Giessen, Licher Straße 62, D-35394 Giessen, Germany

**Keywords:** hate speech, moralized language, social media

## Abstract

Hate speech on social media threatens the mental health of its victims and poses severe safety risks to modern societies. Yet, the mechanisms underlying its proliferation, though critical, have remained largely unresolved. In this work, we hypothesize that moralized language predicts the proliferation of hate speech on social media. To test this hypothesis, we collected three datasets consisting of *N* = 691,234 social media posts and ∼35.5 million corresponding replies from Twitter that have been authored by societal leaders across three domains (politics, news media, and activism). Subsequently, we used textual analysis and machine learning to analyze whether moralized language carried in *source tweets* is linked to differences in the prevalence of hate speech in the corresponding *replies*. Across all three datasets, we consistently observed that higher frequencies of moral and moral-emotional words predict a higher likelihood of receiving hate speech. On average, each additional moral word was associated with between 10.76% and 16.48% higher odds of receiving hate speech. Likewise, each additional moral-emotional word increased the odds of receiving hate speech by between 9.35 and 20.63%. Furthermore, moralized language was a robust out-of-sample predictor of hate speech. These results shed new light on the antecedents of hate speech and may help to inform measures to curb its spread on social media.

Significance Statement This study provides large-scale observational evidence that moralized language fosters the proliferation of hate speech on social media. Specifically, we analyzed three datasets from Twitter covering three domains (politics, news media, and activism) and found that the presence of moralized language in source posts was a robust and meaningful predictor of hate speech in the corresponding replies. These findings offer new insights into the mechanisms underlying the proliferation of hate speech on social media and may help to inform educational applications, counterspeech strategies, and automated methods for hate speech detection.

## Introduction

Social media platforms are a fertile ground for antisocial behavior, including online harassment, cyber-bullying, and, in particular, hate speech (1). Broadly speaking, hate speech refers to abusive or threatening speech (or writing) that attacks a person or group, typically on the basis of attributes such as ethnicity, religion, sex, or sexual orientation (2). Hate speech on social media poses severe risks both to the targeted individuals and society as a whole (2). At the individual level, it threatens the well-being (physically and psychologically) of those affected (1,3). At the societal level, it fosters political polarization (4), which can have severe consequences. Examples include increased opportunities for the spread of misinformation about the target group (5), erosion of existing antidiscriminatory norms (1), and even domestic terrorism (3, 4).

While previous research suggests that hate speech on social media is widespread (6), the mechanisms underlying its proliferation, though critical, have remained largely unresolved. In this work, we approach this question through the lens of morality and its triggering role in social media environments. Social media content delivers not only factual information but also carries moralized content (7). Broadly defined, content is moralized if it references ideas, objects, or events construed in terms of the good of a unit larger than the individual (e.g., society) (8). Since socially connected users often develop similar ideas and intuitions, moralized content is a key driver of information diffusion on social media (7). However, moral ideas have also been postulated to be highly polarizing to social media users (9) and thus might trigger animosity, hostility, and malice from ideologically opposing groups. Prior research has found that moral concerns differentiate hate from dislike (10) and argued that hate may be a response to perceived moral transgressions or wrongdoing of the outgroup (11). In this situation, people may even feel that hurting others is fundamentally right (12). Furthermore, moralized content plays an important role in fulfilling group-identity motives (8) and thus may also trigger hate from ideologically concordant groups rallied up against an outgroup. If moralized content on social media triggers such (negative) reactions in users, then its transmission likely plays a significant role in the proliferation of hate speech. Based on this rationale, we hypothesize that moralized language in social media posts is linked to a higher likelihood of receiving hate speech.

In this study, we investigate the link between moralized language and hate speech on social media. Specifically, we empirically analyze whether differences in the prevalence of hate speech in *replies* to social media posts can be explained by moralized language in the *source post*. To address our research question, we perform a large-scale explanatory analysis based on three datasets consisting of *N* = 691,234 social media posts from Twitter authored by societal leaders across three domains, namely, politics, news media, and activism (see the “Methods” section). We use textual analysis and machine learning to (1) measure moralized language in the source tweets and (2) determine the share of replies to each source tweet that embeds hate speech. Subsequently, we implement multilevel binomial regression models to estimate whether social media users are more likely to receive hate speech if their posts embed moralized language.

## Results

We collected three large-scale datasets consisting of 691,234 source tweets and ∼35.5 million corresponding replies in the domains of politics, news media, and activism (see Supplementary Information, Section 1). Specifically, our dataset contained (i) 335,698 tweets that have been authored by the 532 members of the 117th US Congress, (ii) 307,820 tweets from 635 members of five major US TV news networks (CNN, Fox News, NBC News, CBS News, and ABC News), and (iii) 47,716 tweets from 219 influential activists (climate, animal rights, and LGBTQIA+ activists). For each person in the datasets, we collected *all* tweets (excluding retweets and replies) authored during the entire year of 2021, i.e., within an observation period of 1 year. Politicians were the most active Twitter users, with a monthly average of 52.40 tweets per user. This was followed by newspeople with an average of 40.87 tweets per month and person, and activists with an average of 18.64 tweets per month and person.

**Fig. 1.**
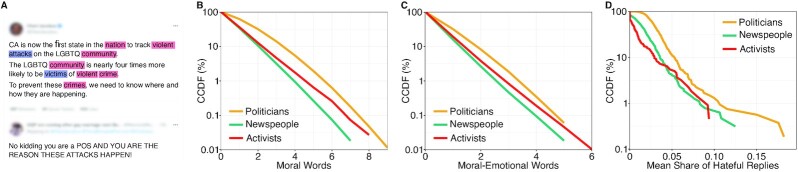
(A) Example of moralized language in a source tweet and hate speech in a reply. Here, moral words are highlighted in blue and moral-emotional words are highlighted in pink. (B and C) Complementary cumulative distribution functions (CCDFs) for the number of moral and moral-emotional words per source tweet. (D) CCDFs showing the mean share of hateful replies individual users received per source tweet.

We studied whether differences in the prevalence of hate speech in replies to tweets can be explained by moralized language carried in the source tweet (see example in Fig. 1A). For this purpose, we first used textual analysis to measure moralized language embedded in the source tweets. Specifically, we employed (and validated) a dictionary-based approach (7) to count the frequencies of occurrence of moral words and moral-emotional words (see the “Methods” section). Politicians tended to use the highest amount of moral and moral-emotional words in their tweets, followed by activists and newspeople (see Fig. 1B and C). Second, we employed (and validated) a machine learning model for hate speech detection (13) in order to identify hate speech in replies to tweets (see the “Methods” section). The hate speech classifier was used to predict a binary label of whether or not a reply tweet is hateful (=1 if true; otherwise =0) for each reply tweet in our data. On average, the share of hateful replies individual users received per source tweet was highest for politicians (3.26%), followed by newspeople (2.11%) and activists (1.61%). Notably, the distributions were right-skewed, indicating that only a small proportion of users received consistently high shares of hateful replies (see Fig. 1D).

Subsequently, we fitted explanatory multilevel binomial regression models to estimate the effects of distinctly moral words and moral-emotional words in source tweets on the likelihood of receiving hate speech in the corresponding replies (see the “Methods” section). In our binomial regression models, the outcome variable was represented as the proportion of hateful replies relative to all replies. We estimated separate models for each of our three datasets and controlled for previously established content variables that may affect the likelihood of receiving hate speech independent of the main predictors (e.g., number of emotional words, word count, and text complexity). The models further included user-specific random effects to control for heterogeneity at the author level (e.g., differences in users’ social influence).

**Fig. 2.**
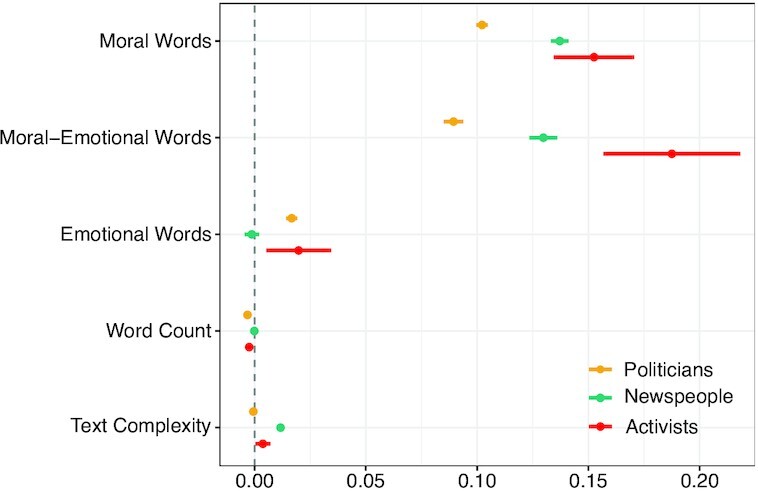
Multilevel binomial regression estimating the effects of moral words, moral-emotional words, and further controls on the likelihood of receiving hate speech. Shown are the coefficient estimates with 99% CIs. User-specific random effects are included.

Figure 2 reports the regression results. Across all three datasets, we consistently observed that higher numbers of both moral words and moral-emotional words in source tweets were linked to a higher likelihood of receiving hate speech in replies. For politicians, each additional moral word was associated with 10.76% higher odds of receiving hate speech (coef = 0.102, 99% CI = [0.100, 0.105], OR = 1.108, *P* < 0.001). Each moral-emotional word increased the odds of receiving hate speech by 9.35% (coef = 0.089, 99% CI = [0.085, 0.094], OR = 1.094, *P* < 0.001). For activists and newspeople, the effects pointed in the same direction. Each additional moral word increased the odds of receiving hate speech of 14.70% for newspeople (coef = 0.137, 99% CI = [0.133, 0.141], OR = 1.147, *P* < 0.001) and 16.48% for activists (coef = 0.153, 99% CI = [0.134, 0.171], OR = 1.165, *P* < 0.001). Each moral-emotional word was linked to an increase in odds of 20.63% for activists (coef = 0.188, 99% CI = [0.157, 0.218], OR = 1.206, *P* < 0.001) and 13.86% for newspeople (coef = 0.130, 99% CI = [0.124, 0.136], OR = 1.139, *P* < 0.001). Linear hypothesis tests implied that the estimates of moral and moral-emotional words were significantly different from each other for politicians (*P* < 0.001), newspeople (*P* = 0.012), and activists (*P* = 0.018).

In sum, across all three datasets, we consistently found that higher frequencies of moral and moral-emotional words in source tweets were linked to more hate speech in the corresponding replies. Notably, the effect sizes of moralized language were fairly pronounced. In comparison, purely emotional words only had negligible positive effects on the likelihood of receiving hate for politicians (coef = 0.017, 99% CI = [0.014, 0.019], OR = 1.017, *P* < 0.001) and activists (coef = 0.020, 99% CI = [0.005, 0.034], OR = 1.020, *P* < 0.001), and were not significant for newspeople (coef = −0.001, 99% CI = [−0.005, 0.002], OR = 0.999, *P* = 0.315). Likewise, the effect sizes of other content characteristics, i.e., the word count (coefs between −0.003 and 0.000; *P* < 0.001 for politicians; *P* < 0.001 for activists; *P* = 0.365 for newspeople) and text complexity (coefs between −0.001 and 0.012; *P* = 0.012 for politicians; *P* < 0.001 for newspeople; *P* = 0.005 for activists) were small. Pairwise comparisons among the coefficient estimates (linear hypothesis tests) confirmed that the estimates of moral and moral-emotional words were significantly greater than for any one of the established content characteristics (all *P* < 0.001).

Multiple exploratory analyses extended our results and confirmed their robustness (see Supplementary Information, Section 5; Tables S1 to S14; Fig. S1). First, we compared our model to an implausible model (14) and tested whether the number of *X*’s, *Y*’s, and *Z*’s in source tweets (i.e., an absurd factor) would have been an equally adequate predictor of hate speech in the replies. Across all three datasets, implausible models resulted in higher AIC values (i.e., lower model adequacy) and effect sizes close to zero. Second, we implemented 10-fold cross-validation to assess the ability of moralized language to predict hate speech prevalence on out-of-sample data. Compared to a baseline model that only used established author and content features, additionally incorporating word counts for moralized language resulted in an out-of-sample *R*^2^ that was 1.22 times higher for politicians, 1.55 times higher for newspeople, and 1.42 times higher for activists. Third, a wide variety of checks confirmed that our findings held for users across both sides of the political spectrum, across different types of hate speech, and when incorporating additional controls (e.g., the retweet count). Taken together, our exploratory analyses provided confirmatory evidence that moralized language was a robust and meaningful predictor of hate speech.

## Discussion

This study provides observational evidence that moralized language in social media posts is associated with more hate speech in the corresponding replies. We uncovered this link for posts from a diverse set of societal leaders across three domains (politics, news media, and activism). On average, each additional moral word was associated with between 10.76 and 16.48% higher odds of receiving hate speech. Likewise, each additional moral-emotional word increased the odds of receiving hate speech by between 9.35 and 20.63%. Across the three domains, the effect sizes were most pronounced for activists. A possible reason is that the activists in our data were affiliated with politically left-leaning subjects (climate, animal rights, and LGBTQIA+) that may have been particularly likely to trigger hate speech from right-wing groups. In contrast, our data for politicians and newspeople were fairly balanced and encompassed users from both sides of the political spectrum. Overall, the comparatively large effect sizes underscore the salient role of moralized language on social media. While earlier research has demonstrated that moralized language is associated with greater virality (7,15), our work implies that it fosters the proliferation of hate speech.

Notably, a connection between morality and hate has been postulated by social psychology theorists for many years, yet empirical evidence has remained scant. Previous work on the psychology of hate and morality argued that hate is rooted in seeing the hated target as morally deficient (11), that morality plays a differentiating role between hate and dislike (10), and that perceptions of outgroup moral wrongdoing may (morally) motivate real-world hate groups (12). Our study adds by demonstrating that moralized language predicts hate speech on social media. Future research may expand upon our work by analyzing users not in a societal leadership role (i.e., regular users), hate speech across ideologically opposing vs concordant groups, and the role of social status in the proliferation of hate speech.

From a practical perspective, observing and understanding the mechanisms underlying the proliferation of hate speech is the first step toward containing it. While we do not advocate that users *should* avoid moralized language in their social media posts, our work still provides a plausible explanation for *why* certain posts/users receive high levels of hate speech. As such, our findings not only help to foster social media literacy but may also inform educational applications, counterspeech strategies, and automated methods for hate speech detection.

## Methods

### Moralized language (Supplementary Information, Section 2)

We applied a dictionary-based approach (7) to count the number of moral, moral-emotional, and emotional words in each source tweet. To validate the [previously validated (7)] dictionaries, we recruited four trained research assistants. Words from the distinctly moral and moral-emotional word lists were rated as more “moral” than words from the distinctly emotional word list and nondictionary words (*P* < 0.001). The annotators yielded a relatively high Kendall’s coefficient of concordance of *W* = 0.67 (*P* = 0.007).

### Hate speech detection (Supplementary Information, Section 3)

We used the dataset from ref. (13) to train a classifier that predicted a binary label of whether a reply was hateful. As validation, two trained research assistants annotated 2,000 reply tweets classified as hateful/not hateful (Kendall’s *W* = 0.69; *P* < 0.001). The classifier achieved a relatively high balanced accuracy of 0.70.

### Model specification (Supplementary Information, Section 4)

We implemented multilevel binomial regressions to estimate the effects of moralized language on the likelihood of receiving hate speech. The number of hate speech replies was modeled as a binomial variable, where the number of trials was given by the total number of replies a tweet received. The key explanatory variables were the absolute counts of moral and moral-emotional words in the source tweets. We controlled for established content characteristics (e.g., emotional words, word count, and text complexity) and used random effects to account for author-level heterogeneity.

## Supplementary Material

pgac281_Supplemental_Files

## Data Availability

Code and anonymized data to replicate the results of this study are available through the Open Science Framework (OSF), https://osf.io/k4baq/.
