# Supplementary material for: Moralized language predicts hate speech on social media
Source: PNAS Nexus. 2022 Dec 7;2(1):pgac281. doi: 10.1093/pnasnexus/pgac281 (PMC9837664; doi:10.1093/pnasnexus/pgac281)
Supplement: pgac281_Supplemental_Files [file pgac281_supplemental_files.zip › PNASNEXUS-PNASNEXUS-2022-00694-s01.pdf]

Supplementary Information for

Moralized language predicts hate speech on social media

Kirill Solovev and Nicolas Pröllochs

Corresponding Author: Nicolas Pröllochs
E-mail: nicolas.proellochs@wi.jlug.de

This PDF file includes:

Supplementary text
Fig. S1
Tables S1 to S14
SI References

Supporting Information Text

1. Data Collection

We collected three large-scale datasets consisting of tweets from societal leaders across three domains:

- *Dataset I (Members of U. S. Congress):* We collected tweets from the 532 members of the 117th U. S. Congress that convened on January 3, 2021. A curated list of Twitter handles of every politician was downloaded from the University of California San Diego library (1). We employed the Twitter API v2 through the Academic Research track (2) to download the complete tweet history (excluding retweets and replies) of each politician between January 3, 2021, and the end of 2021, i. e., for an observation period of approximately one year. The resulting dataset contained 335,698 tweets.

- *Dataset II (Newspeople):* We collected tweets from 635 hosts, regular contributors, anchors, reporters, and correspondents of five major U. S. TV news networks, namely, CNN, Fox News, NBC News, CBS News, and ABC News. The list of newspeople and their Twitter handles was gathered from the webpages of the TV news networks, their social media pages, and via manual web search. We used the Twitter API to download the entire tweet history (excluding retweets and replies) for each person in 2021 i. e., for an observation period of one year. The resulting dataset contained 307,820 tweets.

- *Dataset III (Activists):* We collected tweets from 219 climate, animal rights, and LGBTQIA+ activists. Since we are not aware of a single database for different groups of activists, we employed publicly available lists of activists from Wikipedia and retrieved the corresponding Twitter handles via manual web search. For the 219 activists, we retrieved all 47,716 tweets (excluding retweets and replies) that have been posted during the entire year of 2021.

After collecting the source tweets for each of the three datasets, we queried Twitter's API to gather replies to every source tweet. To ensure computational feasibility, we restricted the data collection to up to 500 replies for each source tweet, starting with the earliest reply. As a check, we also experimented with an alternative variant using random samples of replies. Here, we observed consistent results. Note that there is a possibility that Twitter may have removed some particularly egregious hate speech replies, which were, therefore, not available for our current analyses. In total, our three datasets contained 691,234 source tweets and 35,548,076 replies.

2. Measurement of Moralized Language

We used a dictionary-based approach to measure the extent to which moral language is embedded in the source tweets. For this purpose, we first applied standard preprocessing steps from text mining. Specifically, the running text was converted into lower-case and tokenized, and special characters (e. g., hashtags, emoticons) were removed. Subsequently, we employed the dictionary from (3), which consists of three word lists: (i) a set of distinctly moral words ($N = 343$), (ii) a set of distinctly emotional words ($N = 848$), and (iii) a set of moral-emotional words ($N = 68$) representing words that are both moral and emotional. We used these word lists to calculate the absolute frequencies of moral words, moral-emotional words, and emotional words in each source tweet.

The discriminant validity of each of the three word lists has previously been validated (3). However, as an additional check, we recruited four trained research assistants and repeated the validation procedure. Analogous to the study from (3), participants were presented with words that were randomly sampled from each of the word lists ($n = 40$ for strictly moral words, $n = 40$ for strictly emotional words, and $n = 10$ for moral-emotional words). Additionally, we sampled 40 random words from the Linguistic Inquiry and Word Count (4) that are not present in the aforementioned word lists (i. e., non-dictionary words). The participants were then asked to rate each word on continuous dimensions of morality and emotions. For this, participants had to answer the question "How related is this word to [morality, emotions]" on a 5-point Likert scale ranging from "Completely Unrelated" to "Completely Related".

In our pilot study, words from the distinctly moral and moral-emotional word lists were rated as more "moral" than words from the distinctly emotional word list and non-dictionary words ($P < 0.001$). Words from the distinctly emotional word list were rated as more "emotional" than words from the distinctly moral word list and non-dictionary words ($P < 0.001$). Words from the moral-emotional word list were rated as more "moral" and "emotional" than non-dictionary words ($P < 0.001$). We observed a relatively high Kendall's coefficient of concordance of $W = 0.67$ ($P = 0.007$) for the moral ratings and $W = 0.68$ ($P = 0.004$) for the emotion ratings. Altogether, our pilot study supported the discriminant validity of the dictionaries.

3. Hate Speech Detection

We used machine learning to detect hate speech in replies to tweets. Compared to dictionary-based methods that merely count hate-related words, this approach is generally considered as being more accurate (5).

We implemented machine learning for hate speech detection as follows: we employed the annotated Twitter dataset from (6), containing 25,000 tweets labeled as hateful or not hateful. Each tweet was annotated by at least three users who were explicitly instructed to think about the context of the message and not only the words contained within (6). Purely offensive (non-hateful) language was not considered as hate speech. We used the annotated tweets to implement a deep neural network classifier that predicted whether or not a reply tweet was hateful. Here we used Universal Sentence Encoder (USE) (7) as text representation. The hate speech classifier was used to predict a binary hate speech label (= 1 if true; otherwise = 0) for each reply tweet in our dataset. The machine learning model was implemented in Python 3.8.10 using TensorFlow 2.8.0.

We used a two-pronged approach to evaluate the prediction performance of the machine learning model: (i) we evaluated the out-of-sample prediction performance on the dataset from (6). Here the machine learning classifier yielded an out-of-sample *balanced accuracy* of 0.77 (using 5-fold cross-validation). The predictive performance is similar to previous works (6) and can be seen as reasonably accurate in the context of our study. (ii) We employed two trained research assistants to annotate random subsets of reply tweets that were classified as hateful and not hateful (500 replies per category for each dataset) by the machine learning model. The annotators yielded a Kendall's coefficient of concordance of $W = 0.69$ ($P < 0.001$) and the classifier achieved a *balanced accuracy* of 0.70. This implied that the machine learning classifier was capable of producing relatively reliable hate speech predictions for our data.

4. Regression Analysis

We implemented a multilevel binomial regression to estimate the effects of moralized language of a tweet on receiving hate speech. Formally, we modeled the number of hate speech replies, *HReplies*, as a binomial variable with probability parameter θ. The number of trials was given by the total number of replies a tweet received (*Replies*). The key explanatory variables were the number of moral words (*Moral Words*) and moral-emotional words (*Moral-Emotional Words*). We controlled for the number of purely emotional words (*Emotional Words*), the word count (*Word Count*), and used the Gunning Fog Index (8) as a measure of text complexity (*Text Complexity*). In addition, we used binary variables to control for whether media was attached to the tweet (*Media Attached*; = 1 if true, otherwise 0) and whether the tweet was a quote tweet (*Quote*; = 1 if true, otherwise 0). Based on these variables, we specified the following regression model:

$$\begin{aligned}\text{logit}(\theta) = {} & \beta_0 + \beta_1\, \textit{Moral Words} + \beta_2\, \textit{Moral-Emotional Words} + \beta_3\, \textit{Emotional Words} \\ & + \beta_4\, \textit{Word Count} + \beta_5\, \textit{Text Complexity} + \beta_6\, \textit{Media Attached} + \beta_7\, \textit{Quote} + u_{\text{user}} + \varepsilon,\end{aligned} \quad [1]$$

$$\textit{HReplies} \sim \textit{Binomial}[\textit{Replies}, \theta], \quad [2]$$

with intercept β_0, error term ε, and user-specific random effects u_{user}. Note that the latter was important as it allowed us to control for heterogeneity across users that have authored the source tweets (e. g., varying social influence, different audiences, etc.).

We estimated Eq. 1 and Eq. 2 using MLE and generalized linear models. Our regression analyses were implemented in R 4.2.0 using the `lme4` package (9).

5. Robustness Checks and Exploratory Analyses

We performed a broad set of checks and exploratory analyses to validate the robustness of our findings. In all cases, our results were robust and consistently supported our findings. In the following, we summarize the main results.

A. Variance Inflation Factors. We calculated variance inflation factors for all explanatory variables in our analysis (Table S1). The VIFs ranged from 1.046 to 1.531 and were thus substantially below the critical threshold of five (10). This indicates that multicollinearity was not an issue in our analysis.

B. Alternative Specifications and Estimators. We tested alternative model specifications in which we (i) analyzed ratios of word counts (i. e., word frequencies divided by word counts) instead of word counts (Table S2); (ii) coded variables for moralized language as dichotomous (i. e., with a binary variable indicating whether the source tweet contained one or more moral / moral-emotional words, or none; see Table S3). Furthermore, we repeated our analysis with a zero-one-inflated beta regression that used the share of hateful replies as the dependent variable (Table S4). In all cases, the results were robust and continued to support our findings.

C. Analysis With Interactions. We tested whether the strength of the association between moralized language and hate speech varied depending on the length of the tweet. For this purpose, we extended the regression models from our main analysis with interaction terms between moralized language and the word count (Table S5). The coefficient estimate for the interaction *Moral Words* × *Word Count* was statistically significant and negative for politicians (coef = −0.001, 99% CI = [−0.001, 0.000], OR = 0.999, $P < 0.001$), newspeople (coef = −0.003, 99% CI = [−0.003, −0.002], OR = 0.997, $P < 0.001$), and activists (coef = −0.004, 99% CI = [−0.006, −0.003], OR = 0.996, $P < 0.001$). The coefficient for the interaction *Moral-Emotional Words* × *Word Count* was negative for newspeople (−0.002, 99% CI = [−0.003, −0.002], OR = 0.998, $P < 0.001$) and activists (coef = −0.005, 99% CI = [−0.007, −0.002], OR = 0.995, $P < 0.001$). We observed no statistically significant coefficient for politicians (coef = −0.000, 99% CI=[−0.001, 0.000], OR = -0.000, $P = 0.254$). Overall, these results suggested that the link between moralized language and hate speech tended to be (slightly) stronger for shorter source tweets.

D. Directed vs. Generalized Hate Speech. We explored whether the link between moralized language and hate speech differed across different types of hate speech. Hate speech can be directed at a specific user (directed hate speech) or at a general group of individuals (generalized hate speech) (11). Previous research (11) has shown that directed hate speech is correlated with higher use of second-person pronouns (e. g., *you*, *your*), whereas generalized hate speech is correlated with higher use of third-person plural pronouns (e. g., *they*, *themselves*). We thus employed the LIWC dictionary (4) to identify the presence of

second-person pronouns (as a proxy for directed hate speech) and third-person plural pronouns (as a proxy for generalized hate speech) in each hateful reply in our datasets. Second-person pronouns were present in 68% of the hateful replies to source tweets from politicians, in 41% of the hateful replies to source tweets from newspeople, and in 53% of the hateful replies to source tweets from activists. For third-person plural pronouns, these numbers amounted to 10% for politicians, 15% for newspeople, and 13% for activists. Thus, across all three datasets, directed hate speech was more prevalent than generalized hate speech.

Subsequently, we repeated our analysis with a regression model in which we replaced *HReplies* (i. e., the number of hateful replies) with count variables that measured the number of directed hate speech replies (i. e., the number of replies that were both hateful and contained second-person pronouns) and generalized hate speech replies (i. e., the number of replies that were both hateful and contained third-person plural pronouns). The regression results are reported in Table S6. Across all three datasets, the effect sizes of moralized language were larger for generalized hate speech than for directed hate speech. For politicians, the coefficient of *Moral Words* was 0.162 (99% CI = [0.155, 0.170], OR = 1.176, $P < 0.001$) for generalized hate speech and 0.092 (99% CI = [0.089, 0.095], OR = 1.097, $P < 0.001$) for directed hate speech. For newspeople, the coefficient of *Moral Words* was 0.187 (99% CI = [0.177, 0.196], OR = 1.205, $P < 0.001$) for generalized hate speech and 0.105 (99% CI = [0.099, 0.111], OR = 1.111, $P < 0.001$) for directed hate speech. For activists, the coefficient of *Moral Words* was 0.202 (99% CI = [0.159, 0.246], OR = 1.224, $P < 0.001$) for generalized hate speech and 0.145 (99% CI = [0.121, 0.170], OR = 1.156, $P < 0.001$) for directed hate speech. The patterns were similar for moral-emotional words. For politicians, the coefficient of *Moral-Emotional Words* was 0.136 (99% CI = [0.123, 0.149], OR = 1.146, $P < 0.001$) for generalized hate speech and 0.088 (99% CI = [0.083, 0.094], OR = 1.092, $P < 0.001$) for directed hate speech. For newspeople, the coefficient of *Moral-Emotional Words* was 0.158 (99% CI = [0.143, 0.173], OR = 1.171, $P < 0.001$) for generalized hate speech and 0.107 (99% CI = [0.098, 0.117], OR = 1.113, $P < 0.001$) for directed hate speech. For activists, the coefficient of *Moral-Emotional Words* was 0.238 (99% CI = [0.161, 0.314], OR = 1.269, $P < 0.001$) for generalized hate speech and 0.174 (99% CI = [0.132, 0.215], OR = 1.190, $P < 0.001$) for directed hate speech.

In sum, across all three datasets, moralized language predicted both directed and generalized hate speech (with larger effect sizes for generalized hate speech). These findings add to the validity of our results.

E. Positive and Negative Emotions in Source Tweets. The variable *Emotional Words* in our main analysis measured the total number of distinctly positive and negative emotion words in the source tweets (see (3)). As a check, we used the LIWC dictionary (4) to measure the number of distinctly positive and negative words separately; and repeated our regression analysis. The regression results are reported in Table S7. For every distinctly negative emotional word, the odds of receiving hate speech were 4.65% higher for politicians (coef = 0.045, 99% CI=[0.041, 0.050], OR = 1.047, $P < 0.001$), 4.49% higher for newspeople (coef = 0.044, 99% CI=[0.039, 0.049], OR = 1.045, $P < 0.001$), and 12.18% higher for activists (coef = 0.115, 99% CI=[0.091, 0.139], OR = 1.122, $P < 0.001$). For every distinctly positive emotional word, the odds of receiving hate speech were 2.56% lower for newspeople (coef = -0.026, 99% CI=[-0.031, -0.021], OR = 0.974, $P < 0.001$), 3.34% lower for activists (coef = -0.034, 99% CI=[$-0.0.54$, -0.014], OR = 0.967, $P < 0.001$), and 0.26% higher (coef = 0.003, 99% CI=[-0.001, 0.006], OR = 1.003, $P = 0.042$) for politicians. Overall, these findings indicate that a more negative sentiment in the source tweets was linked to more hate speech in the replies. All findings for moralized language remained robust.

F. Hate Speech in Source Tweets. We tested whether hate speech in the source tweets was linked to more hate speech in the corresponding replies. For this purpose, we used our machine learning classifier to predict a binary hate speech label (= 1 if true; otherwise = 0) for each source tweet in our dataset. We then repeated our regression analysis with this additional explanatory variable (see Table S8). The coefficient for *Hateful Source* was positive and statistically significant for politicians (coef = 0.734, OR = 2.082, $P < 0.001$, 99% CI=[0.713, 0.754], newspeople (coef = 1.018, 99% CI=[1.001, 1.036], OR = 2.769, $P < 0.001$), and activists (coef = 0.905, 99% CI=[0.813, 0.998], OR = 2.472, $P < 0.001$). This indicated that hate speech in source tweets was linked to more hate speech in the corresponding replies. All findings for moralized language remained robust.

G. Engagement With Source Tweets. We constructed additional regression to control for the virality / the level of engagement with the source tweet. Specifically, we implemented two model variants that either used the number of retweets or the number of likes as an additional explanatory variable. Note that we used separate regression models as both variables were highly correlated (correlation of 0.895, 0.886, and 0.880 for politicians, newspeople, and activists, respectively). Across all datasets, the coefficient estimates for the number of retweets (Table S9) and the number of likes (Table S10) were positive and statistically significant. However, the effect sizes for both the number of retweets (standardized coefficients between 0.007 and 0.022) and the number of likes (standardized coefficients between 0.008 and 0.017) were rather small. All findings for moralized language remained robust.

H. Comprehensive Model With All Additional Control Variables. We estimated a comprehensive regression model with all additional control variables from our exploratory analysis. The results are reported in Table S11. All findings for moralized language remained robust.

I. Analysis of Political Alignment. Our data for politicians (Dataset I) and newspeople (Dataset II) encompassed users from both sides of the political spectrum. This allowed us to test how the strength of association between moralized language and hate speech varied for people from both political parties and across different political leanings of the TV news networks. For this purpose, we implemented two additional regression models: (i) for politicians, we added interaction terms between the

predictors for moralized language and the political party of the author of the source tweet (=1 if Democratic; =0 if Republican); (ii) for newspeople, we added interaction terms between the predictors for moralized language and the political leaning of the TV news networks (i.e. *Left*, *Center* or *Right*). To determine the political leanings of the TV news networks, we utilized the website https://mediabiasfactcheck.com, which provides assessment of political leanings for a large number of media sources. This resulted in the following categorizations of political leanings: *Left* for CNN; *Center* for ABC News, CBS News, and NBC News; *Right* for Fox News.

The regression results are reported in Table S12. For politicians, the coefficient estimate for the interactions between party affiliation and the predictors for moralized language were statistically significant and positive. The coefficient estimate for *Moral Words* × *Democratic* was 0.008 (99% CI=[0.003, 0.014], OR = 1.009, $P < 0.001$) and the coefficient estimate for the interaction *Moral-Emotional Words* × *Democratic* was 0.041 (99% CI=[0.032, 0.050], OR = 1.042, $P < 0.001$). At the same time, the coefficients of the direct effects of *Moral Words* and *Moral-Emotional Words* remained statistically significant and similar in magnitude as in our main analysis. These results implied that the link between moralized language and hate speech held for both sides of the political spectrum. However, tweets authored by politicians from the Democratic Party were (slightly) more likely to receive hateful replies in response to moralized language than Republicans.

We observed a similar pattern for the dataset with newspeople (Table S12). The coefficient estimate for the interaction *Moral Words*× *Right* was 0.016 (99% CI=[0.005, 0.0027], OR = 1.016, $P < 0.001$) and the coefficient estimate for the interaction *Moral Words* × *Left* was 0.017 (99% CI=[0.005, 0.029], OR = 1.017, $P < 0.001$). The coefficient estimate for the interaction *Moral-Emotional Words* × *Right* was 0.019 (99% CI=[0.001, 0.037], OR = 0.019, $P = 0.007$) and the coefficient estimate for *Moral-Emotional Words* × *Left* lacked statistical significance (coef = 0.008, 99% CI=[−0.012, 0.028], OR = 1.008, $P = 0.284$). At the same time, the direct effects of *Moral Words* and *Moral-Emotional Words* remained statistically significant and similar in magnitude as in our main analysis. These findings implied that the link between moralized language and hate speech held across all political leanings of TV news networks. The differences in the effect sizes between left-leaning vs. right-leaning TV news networks were rather small. However, tweets from newspeople affiliated with left-leaning and right-leaning TV news networks were significantly more likely to receive hate speech in response to moralized language than those from newspeople affiliated with center-oriented TV news networks.

We further note that both models in Table S12 showed statistically significant estimates for the direct effect of the political leanings on the likelihood of receiving hate speech. All else being equal, users affiliated with a right-leaning political party (i. e., Republicans) or TV news network (i. e., Fox News) were more likely to receive hate speech on social media.

Taken together, we found that the hypothesized link between moralized language and hate speech was generalizable to both sides of the political spectrum.

J. Comparison to an Implausible XYZ Model. Prior research noted that large-scale observational studies can sometimes yield fragile results and even support patently absurd models (12). Following earlier work, we thus compared our model to an implausible XYZ model (12). Specifically, we counted the number of X's, Y's and Z's in source tweets (i. e., an absurd factor) and tested whether this variable (*XYZ Count*) would have been an equally adequate predictor of hate speech replies. The regression results are reported in Table S13. Across all three datasets, XYZ models resulted in higher AIC values (i. e., lower model adequacy) and effect sizes close to zero. The coefficient of *XYZ Count* was 0.009 ($P < 0.001$) for politicians and −0.015 ($P < 0.001$) for activists. For newspeople, the coefficient of *XYZ Count* was not statistically significant ($P = 0.304$). For comparison, the coefficient of *Moral Words* was 0.102 for politicians ($P < 0.001$), 0.137 for newspeople ($P < 0.001$), and 0.153 ($P < 0.001$) for activists. These findings provided strong evidence that moralized language was a meaningful predictor of hate speech.

K. Out-of-Sample Evaluation. We used 10-fold cross-validation to assess the ability of moralized language to predict hate speech prevalence on out-of-sample data. For this purpose, we implemented multiple binomial regression models that took different sets of predictors into account. This approach allowed us to compare the out-of-sample prediction performance across different predictor sets. The individual predictor sets were as follows: (i) author features* (the number of followers, the number of followees, the account age, and the verified status), (ii) established content features (*Word Count*, *Text Complexity*, and *Emotional Words*, *Quote*, *Media Attached*), and (iii) word counts for moralized language (*Moral Words*, *Moral-Emotional Words*). Note that we used the same linear model as in our in-sample analysis but replaced the author-specific random effect terms with common author-specific variables from previous work. This ensures that out-of-sample predictions can be made for users not present in the training data. All models were implemented in R 4.2.0 using the `tidymodels` package (13).

The prediction results for different feature combinations are reported in Table S14. Since we used linear regression models, we report the prediction performance in terms of out-of-sample R^2 (calculated using 10-fold cross-validation). Compared to a baseline model that used only established author and content features, additionally incorporating word counts for moralized language resulted in an out-of-sample R^2 that was 1.26 times, 1.57 times, and 1.42 times higher for politicians, newspeople, and activists, respectively.

While our study focuses on predicting the frequency of hateful replies (i. e., a regression problem), we also tested a variant in which we treated the task of identifying source tweets with a disproportionally high share of hateful replies as a binary classification problem. Specifically, we used a logistic regression model and defined a binary response variable *ExtremelyHateful*, which took the value = 1 for source tweets that received a disproportionally high share of hateful replies (otherwise = 0). In

*All author variables were retrieved from the Twitter API v2.

each dataset, source tweets with the 25% highest share of hateful replies were considered as being *ExtremelyHateful*. To ensure that the analysis was not driven by outliers (e. g., tweets that received only one hateful reply), we excluded source tweets that have received less than 10 total replies. The out-of-sample ROC curves and ROC-AUC (calculated via 10-fold cross-validation) across different sets of predictors are reported in Figure S1. We again observed that moralized language was a meaningful predictor of hate speech. Compared to a baseline model that used only established author and content features, additionally incorporating word counts for moralized language resulted in a ROC-AUC that was 6.62% higher for politicians, 4.48% higher for newspeople, and 2.77% higher for activists. Delongs's tests confirmed that the differences in ROC-AUC between the models w/ and w/o features for moralized language were statistically significant (all $P < 0.001$).

As an additional check, we counted the number of X's, Y's and Z's in source tweets (i. e., an absurd predictor) and tested whether this variable (*XYZ Count*) enhances the prediction performance. Consistent with our in-sample analysis, including *XYZ Count* as an additional predictor resulted in practically no changes in out-of-sample R^2 and ROC-AUC. In sum, our out-of-sample analysis confirmed that moralized language was a robust and meaningful predictor of hate speech. Future research may expand on these results by implementing (non-linear) machine learning models for predicting hate speech prevalence.

6. Ethics

This research uses public tweets only, and, thus, no approval from the Institutional Review Board was required by the authors' institutions.

Table S1. Variance inflation factors.

	Politicians	Newspeople	Activists
Moral Words	1.153	1.143	1.155
Moral-Emotional Words	1.057	1.063	1.084
Emotional Words	1.151	1.198	1.225
Word Count	1.474	1.415	1.531
Text Complexity	1.184	1.050	1.120
Media Attached	1.055	1.055	1.094
Quote	1.046	1.072	1.154

Table S2. Regression results with proportions of word counts (i. e., word frequencies divided by word counts).

	Politicians	Newspeople	Activists
Moral Words	2.625***	3.398***	3.251***
	(0.028)	(0.037)	(0.132)
Moral-Emotional Words	2.111***	2.973***	4.665***
	(0.046)	(0.059)	(0.268)
Emotional Words	0.363***	−0.134***	0.689***
	(0.025)	(0.031)	(0.146)
Word Count	0.002***	0.005***	0.004***
	(0.000)	(0.000)	(0.001)
Text Complexity	−0.001***	0.012***	0.005***
	(0.000)	(0.000)	(0.001)
Media Attached (binary)	0.054***	−0.008	−0.118***
	(0.003)	(0.005)	(0.019)
Quote (binary)	0.046***	0.127***	0.165***
	(0.004)	(0.004)	(0.020)
Intercept	−3.568***	−4.471***	−5.083***
	(0.021)	(0.035)	(0.089)
Random effects (user level)	Included	Included	Included
Observations	335,698	307,820	47,716
AIC	806,293	765,918	48,591

Significance levels: $^{*}p < 0.05$, $^{**}p < 0.01$, $^{***}p < 0.001$; standard errors in parentheses

Table S3. Regression results with binary variables for moral and moral-emotional language (=1 if the tweet contained one or more moral / moral-emotional words, otherwise 0).

	Politicians	Newspeople	Activists
Moral Words (binary)	0.168***	0.236***	0.321***
	(0.003)	(0.003)	(0.016)
Moral-Emotional Words (binary)	0.131***	0.189***	0.265***
	(0.003)	(0.004)	(0.018)
Emotional Words	0.020***	0.000	0.020***
	(0.001)	(0.001)	(0.006)
Word Count	−0.002***	0.001***	−0.002***
	(0.000)	(0.000)	(0.001)
Text Complexity	0.000	0.012***	0.004***
	(0.000)	(0.000)	(0.001)
Media Attached (binary)	0.046***	−0.011*	−0.124***
	(0.003)	(0.005)	(0.019)
Quote (binary)	0.039***	0.121***	0.146***
	(0.004)	(0.004)	(0.020)
Intercept	−3.483***	−4.395***	−4.962***
	(0.021)	(0.035)	(0.088)
Random effects (user level)	Included	Included	Included
Observations	335,698	307,820	47,716
AIC	809,386	767,155	48,591

Significance levels: $^{*}p < 0.05$, $^{**}p < 0.01$, $^{***}p < 0.001$; standard errors in parentheses

Table S4. Coefficient estimates for zero-one-inflated beta regression. The dependent variable is the share of hateful replies.

	Politicians	Newspeople	Activists
Moral Words	0.058***	0.070***	0.071***
	(0.002)	(0.003)	(0.011)
Moral-Emotional Words	0.068***	0.072***	0.073***
	(0.003)	(0.005)	(0.017)
Emotional Words	0.038***	−0.006**	−0.003
	(0.002)	(0.002)	(0.008)
Word Count	−0.003***	−0.003***	−0.003**
	(0.000)	(0.000)	(0.001)
Text Complexity	0.000	0.011***	0.008***
	(0.000)	(0.001)	(0.002)
Media Attached (binary)	0.073***	−0.025**	−0.079**
	(0.005)	(0.009)	(0.027)
Quote (binary)	0.096***	0.178***	0.247***
	(0.006)	(0.007)	(0.029)
Intercept	−2.155***	−2.348***	−2.339***
	(0.029)	(0.035)	(0.094)
Random effects (user level)	Included	Included	Included
Observations	335,698	307,820	47,716
WAIC	−44,240	−75,268	−4890

significance levels: $^{*}p < 0.05$, $^{**}p < 0.01$, $^{***}p < 0.001$; standard errors in parentheses

Table S5. Regression results with interactions between moralized language and word count.

	Politicians	Newspeople	Activists
Moral Words × Word Count	−0.001***	−0.003***	−0.004***
	(0.000)	(0.000)	(0.001)
Moral-Emotional Words × Word Count	0.000	−0.002***	−0.005***
	(0.000)	(0.000)	(0.001)
Moral Words	0.123***	0.248***	0.332***
	(0.003)	(0.004)	(0.025)
Moral-Emotional Words	0.096***	0.211***	0.385***
	(0.006)	(0.007)	(0.040)
Emotional Words	0.017***	−0.001	0.020***
	(0.001)	(0.001)	(0.006)
Word Count	−0.003***	0.002***	0.000
	(0.000)	(0.000)	(0.001)
Text Complexity	−0.001**	0.011***	0.004**
	(0.000)	(0.000)	(0.001)
Media Attached (binary)	0.053***	−0.003	−0.123***
	(0.003)	(0.005)	(0.019)
Quote (binary)	0.041***	0.128***	0.160***
	(0.004)	(0.004)	(0.020)
Intercept	−3.458***	−4.386***	−5.007***
	(0.021)	(0.034)	(0.088)
Random effects (user level)	Included	Included	Included
Observations	335,698	307,820	47,716
AIC	803,165	764,419	48,387

Significance levels: $^{*}p < 0.05$, $^{**}p < 0.01$, $^{***}p < 0.001$; standard errors in parentheses

Table S6. Regression results for predicting the number of hateful replies that include second-person pronouns (2PP) and third-person plural pronouns (3PP). Second person pronouns tend to occur in conjunction with directed hate speech (i. e., hate against a specific person), whereas third person plural pronouns tend to occur in conjunction with generalized hate speech (i. e., hate against a group) (11).

	Politicians		Newspeople		Activists	
	2PP	3PP	2PP	3PP	2PP	3PP
Moral Words	0.092***	0.162***	0.105***	0.187***	0.145***	0.202***
	(0.001)	(0.003)	(0.002)	(0.004)	(0.010)	(0.017)
Moral-Emotional Words	0.088***	0.136***	0.107***	0.158***	0.174***	0.238***
	(0.002)	(0.005)	(0.004)	(0.006)	(0.016)	(0.030)
Emotional Words	0.022***	0.025***	0.023***	−0.025***	0.035***	0.029*
	(0.001)	(0.003)	(0.002)	(0.003)	(0.008)	(0.015)
Word Count	−0.002***	−0.004***	0.004***	0.000	0.000	−0.001
	(0.000)	(0.000)	(0.000)	(0.000)	(0.001)	(0.002)
Text Complexity	−0.002***	0.003***	0.007***	0.017***	−0.003	0.011**
	(0.000)	(0.001)	(0.000)	(0.001)	(0.002)	(0.004)
Media Attached (binary)	0.063***	0.021*	0.060***	−0.082***	−0.113***	−0.282***
	(0.003)	(0.009)	(0.007)	(0.012)	(0.025)	(0.052)
Quote (binary)	−0.007	0.076***	0.110***	0.086***	0.133***	0.183***
	(0.005)	(0.011)	(0.006)	(0.010)	(0.028)	(0.054)
Intercept	−3.889***	−5.758***	−5.394***	−6.255***	−5.682***	−7.130***
	(0.024)	(0.026)	(0.040)	(0.040)	(0.101)	(0.139)
Random effects (user level)	Included	Included	Included	Included	Included	Included
Observations	335,698	335,698	307,820	307,820	47,716	47,716
AIC	642,572	247,116	414,477	260,770	29,111	13,961

Significance levels: $^{*}p < 0.05$, $^{**}p < 0.01$, $^{***}p < 0.001$; standard errors in parentheses

Table S7. Regression results with distinctly positive and distinctly negative emotional words.

	Politicians	Newspeople	Activists
Moral Words	0.102***	0.135***	0.145***
	(0.001)	(0.002)	(0.007)
Moral-Emotional Words	0.089***	0.127***	0.178***
	(0.002)	(0.002)	(0.012)
Positive Emotional Words	0.003*	−0.026***	−0.034***
	(0.001)	(0.002)	(0.008)
Negative Emotional Words	0.045***	0.044***	0.115***
	(0.002)	(0.002)	(0.009)
Word Count	−0.003***	0.000**	−0.002**
	(0.000)	(0.000)	(0.001)
Text Complexity	−0.001***	0.011***	0.003*
	(0.000)	(0.000)	(0.001)
Media Attached (binary)	0.056***	−0.004	−0.133***
	(0.003)	(0.005)	(0.019)
Quote (binary)	0.040***	0.120***	0.163***
	(0.004)	(0.004)	(0.020)
Intercept	−3.443***	−4.341***	−4.886***
	(0.021)	(0.034)	(0.087)
Random effects (user level)	Included	Included	Included
Observations	335,698	307,820	47,716
AIC	802,699	764,622	48,318

Significance levels: $^{*}p < 0.05$, $^{**}p < 0.01$, $^{***}p < 0.001$; standard errors in parentheses

Table S8. Regression results controlling for hate speech in the source tweet (*Hateful Source*).

	Politicians	Newspeople	Activists
Moral Words	0.097***	0.121***	0.139***
	(0.001)	(0.002)	(0.007)
Moral-Emotional Words	0.085***	0.116***	0.174***
	(0.002)	(0.002)	(0.012)
Emotional Words	0.017***	−0.001	0.018**
	(0.001)	(0.001)	(0.006)
Word Count	−0.003***	0.001***	−0.002*
	(0.000)	(0.000)	(0.001)
Text Complexity	−0.001**	0.011***	0.004**
	(0.000)	(0.000)	(0.001)
Media Attached (binary)	0.053***	−0.014**	−0.120***
	(0.003)	(0.005)	(0.019)
Quote (binary)	0.033***	0.108***	0.145***
	(0.004)	(0.004)	(0.020)
Hateful Source (binary)	0.734***	1.018***	0.905***
	(0.008)	(0.007)	(0.036)
Intercept	−3.467***	−4.362***	−4.935***
	(0.021)	(0.034)	(0.086)
Random effects (user level)	Included	Included	Included
Observations	335,698	307,820	47,716
AIC	796,188	747,931	47,953

Significance levels: $^{*}p < 0.05$, $^{**}p < 0.01$, $^{***}p < 0.001$; standard errors in parentheses

Table S9. Regression results controlling for the number of retweets of the source tweet. Due to varying scales, all numeric variables have been standardized.

	Politicians	Newspeople	Activists
Moral Words	0.128***	0.112***	0.142***
	(0.001)	(0.001)	(0.007)
Moral-Emotional Words	0.064***	0.062***	0.100***
	(0.001)	(0.001)	(0.007)
Emotional Words	0.024***	−0.001	0.025**
	(0.001)	(0.002)	(0.008)
Word Count	−0.039***	−0.003	−0.034***
	(0.001)	(0.002)	(0.010)
Text Complexity	−0.003*	0.069***	0.025**
	(0.001)	(0.002)	(0.008)
Media Attached (binary)	0.055***	−0.003	−0.140***
	(0.003)	(0.005)	(0.019)
Quote (binary)	0.046***	0.137***	0.165***
	(0.004)	(0.004)	(0.020)
Retweet Count	0.007***	0.017***	0.022***
	(0.000)	(0.001)	(0.002)
Intercept	−3.400***	−4.125***	−4.777***
	(0.021)	(0.034)	(0.085)
Random effects (user level)	Included	Included	Included
Observations	335,698	307,820	47,716
AIC	802,908	764,330	48,380

Significance levels: $^{*}p < 0.05$, $^{**}p < 0.01$, $^{***}p < 0.001$; standard errors in parentheses

Table S10. Regression results controlling for the number of likes of the source tweet. Due to varying scales, all numeric variables have been standardized.

	Politicians	Newspeople	Activists
Moral Words	0.128***	0.112***	0.143***
	(0.001)	(0.001)	(0.007)
Moral-Emotional Words	0.064***	0.062***	0.103***
	(0.001)	(0.001)	(0.007)
Emotional Words	0.023***	−0.002	0.025**
	(0.001)	(0.002)	(0.008)
Word Count	−0.037***	−0.001	−0.034***
	(0.001)	(0.002)	(0.010)
Text Complexity	−0.003*	0.070***	0.026**
	(0.001)	(0.002)	(0.008)
Media Attached (binary)	0.055***	−0.006	−0.143***
	(0.003)	(0.005)	(0.019)
Quote (binary)	0.047***	0.129***	0.163***
	(0.004)	(0.004)	(0.020)
Like Count	0.008***	0.008***	0.017***
	(0.000)	(0.001)	(0.002)
Intercept	−3.400***	−4.117***	−4.774***
	(0.021)	(0.034)	(0.085)
Random effects (user level)	Included	Included	Included
Observations	335,698	307,820	47,716
AIC	802,824	765,146	48,423

Significance levels: $^{*}p < 0.05$, $^{**}p < 0.01$, $^{***}p < 0.001$; standard errors in parentheses

Table S11. Comprehensive regression model with all additional control variables. Due to varying scales, all numeric variables have been standardized.

	Politicians	Newspeople	Activists
Moral Words × WordCount	−0.004**	−0.028***	−0.056***
	(0.001)	(0.001)	(0.008)
Moral-Emotional Words × WordCount	−0.002	−0.014***	−0.034***
	(0.001)	(0.001)	(0.008)
Moral Words	0.122***	0.116***	0.174***
	(0.001)	(0.002)	(0.010)
Moral-Emotional Words	0.061***	0.065***	0.111***
	(0.001)	(0.001)	(0.009)
Positive Emotional Words	0.005***	−0.020***	−0.030***
	(0.001)	(0.002)	(0.008)
Negative Emotional Words	0.031***	0.026***	0.071***
	(0.001)	(0.001)	(0.007)
Word Count	−0.035***	0.009***	−0.026**
	(0.001)	(0.002)	(0.010)
Text Complexity	−0.005***	0.062***	0.018*
	(0.001)	(0.002)	(0.008)
Media Attached (binary)	0.059***	0.002	−0.094***
	(0.003)	(0.005)	(0.019)
Quote (binary)	0.039***	0.133***	0.172***
	(0.004)	(0.004)	(0.020)
Hateful Source (binary)	0.726***	1.006***	0.847***
	(0.008)	(0.007)	(0.036)
Retweet Count	0.007***	0.017***	0.019***
	(0.000)	(0.001)	(0.002)
Intercept	−3.413***	−4.124***	−4.787***
	(0.020)	(0.034)	(0.083)
Random effects (user level)	Included	Included	Included
AIC	795,521	745,749	47,687
Observations	335,698	307,820	47,716

Significance levels: $^{*}p < 0.05$, $^{**}p < 0.01$, $^{***}p < 0.001$; standard errors in parentheses

Table S12. Regression results with interactions between moralized language and political leanings. For politicians, the binary variable *Democratic* indicates whether a politician is affiliated with the Democratic Party (=1 if true, otherwise 0). The reference category refers to politicians affiliated with the Republican Party. For newspeople, the binary variables *Right* and *Left* indicate whether a newsperson is affiliated with a left-leaning or right-leaning TV news network. The reference category refers to newspeople affiliated with center-oriented TV news networks.

	Politicians	Newspeople
Moral Words × Democratic	0.008***	
	(0.002)	
Moral-Emotional Words × Democratic	0.041***	
	(0.003)	
Moral Words × Right		0.016***
		(0.004)
Moral Words × Left		0.017***
		(0.005)
Moral-Emotional Words × Right		0.019**
		(0.007)
Moral-Emotional Words × Left		0.008
		(0.008)
Moral Words	0.100***	0.123***
	(0.001)	(0.004)
Moral-Emotional Words	0.074***	0.117***
	(0.002)	(0.006)
Emotional Words	0.017***	−0.001
	(0.001)	(0.001)
Word Count	−0.003***	0.000
	(0.000)	(0.000)
Text Complexity	−0.001*	0.012***
	(0.000)	(0.000)
Media Attached (binary)	0.052***	−0.009
	(0.003)	(0.005)
Quote (binary)	0.040***	0.120***
	(0.004)	(0.004)
Democratic (binary)	−0.276***	
	(0.040)	
Right (binary)		0.367***
		(0.090)
Left (binary)		0.120
		(0.074)
Intercept	−3.318***	−4.459***
	(0.028)	(0.050)
Random effects (user level)	Included	Included
Observations	335,698	307,820
AIC	803,007	765,301

Significance levels: $^{*}p < 0.05$, $^{**}p < 0.01$, $^{***}p < 0.001$; standard errors in parentheses

Table S13. Comparison to an implausible XYZ model (12). The variable *XYZ Count* measures the number of X's, Y's and Z's in the source tweets (i. e., an absurd factor).

	Politicians		Newspeople		Activists	
	XYZ	Main	XYZ	Main	XYZ	Main
Moral Words		0.102***		0.137***		0.153***
		(0.001)		(0.002)		(0.007)
Moral-Emotional Words		0.089***		0.130***		0.188***
		(0.002)		(0.002)		(0.012)
Emotional Words	0.024***	0.017***	0.002	−0.001	0.028***	0.020***
	(0.001)	(0.001)	(0.001)	(0.001)	(0.006)	(0.006)
Word Count	0.000	−0.003***	0.004***	0.000	0.004***	−0.002***
	(0.000)	(0.000)	(0.000)	(0.000)	(0.001)	(0.001)
Text Complexity	0.001***	−0.001*	0.013***	0.012***	0.005***	0.004**
	(0.000)	(0.000)	(0.000)	(0.000)	(0.001)	(0.001)
Media Attached (binary)	0.032***	0.052***	−0.036***	−0.009	−0.144***	−0.142***
	(0.003)	(0.003)	(0.005)	(0.005)	(0.019)	(0.019)
Quote (binary)	0.029***	0.040***	0.110***	0.120***	0.162***	0.153***
	(0.004)	(0.004)	(0.004)	(0.004)	(0.020)	(0.020)
XYZ Count	0.009***		0.001		−0.015***	
	(0.001)		(0.001)		(0.004)	
Intercept	−3.462***	−3.448***	−4.387***	−4.352***	−4.923***	−4.899***
	(0.021)	(0.021)	(0.035)	(0.035)	(0.089)	(0.087)
Random effects (user level)	Included	Included	Included	Included	Included	Included
Observations	335,698	335,698	307,820	307,820	47,716	47,716
AIC	816,067	803,205	775,894	765,333	49,232	48,474

Significance levels: $^{*}p < 0.05$, $^{**}p < 0.01$, $^{***}p < 0.001$; standard errors in parentheses

Table S14. Out-of-sample R^2 (calculated via 10-fold cross-validation) for different sets of predictors. Author features include common author-specific predictors from previous work (i. e., the number of followers, the number of followees, the account age, and the verified status). Content features include established text characteristics (*Word Count*, *Text Complexity*, *Emotional Words*, *Quote*, and *Media Attached*). The predictors for moralized language include the moral and moral-emotional word counts. The variable *XYZ Count* measures the number of X's, Y's and Z's in source tweets (i. e., an implausible predictor).

Predictors	Politicians	Newspeople	Activists
Author Variables	0.029	0.033	0.065
Author Variables + Content Variables	0.047	0.039	0.077
Author Variables + Content Variables + XYZ Count	0.047	0.039	0.076
Author Variables + Content Variables + Moralized Language	0.059	0.060	0.110

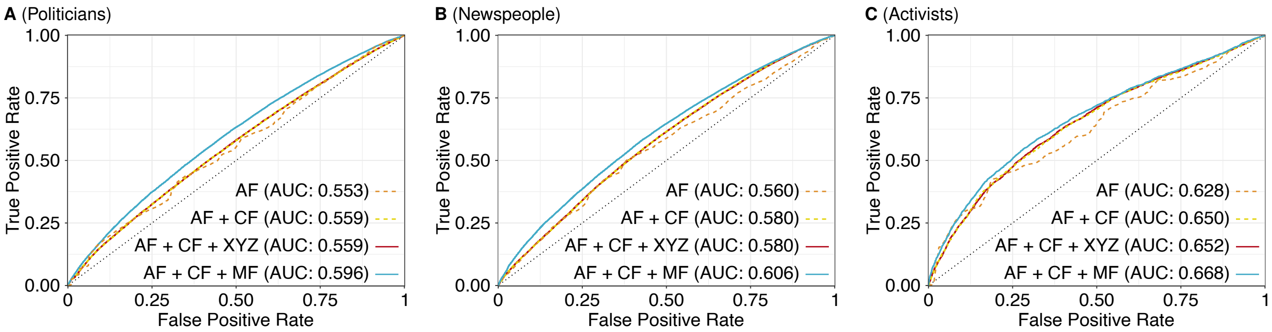

Fig. S1. Out-of-sample ROC curves and ROC-AUC (calculated via 10-fold cross-validation) across different sets of predictors for (A) Politicians, (B) Newspeople, and (C) Activists. Here, we treated the task of predicting hate speech in replies to source tweets as a binary classification problem. For this, we defined a response variable *ExtremelyHateful*, which took the value $= 1$ for source tweets that received a disproportionally high share of hateful replies (otherwise $= 0$). In each dataset, source tweets with the 25% highest share of hateful replies were considered as being *ExtremelyHateful*. Source tweets that have received less than 10 replies were excluded. Author features (*AF*) include common author-specific predictors from previous work (i. e., the number of followers, the number of followees, the account age, and the verified status). Content features (*CF*) include established text characteristics (*Word Count*, *Text Complexity*, *Emotional Words*, *Quote*, and *Media Attached*). The predictors for moralized language (*MF*) include the moral and moral-emotional word counts. The predictor *XYZ* measures the number of X's, Y's and Z's in source tweets (i. e., an implausible predictor).

References

1. Kelly L. Smith. LibGuides: Congressional Twitter accounts, 2021. URL https://ucsd.libguides.com/congress_twitter.
2. Twitter. Twitter API v2, 2022. URL https://developer.twitter.com/en/docs/twitter-api.
3. William J Brady, Julian A Wills, John T Jost, Joshua A Tucker, and Jay J Van Bavel. Emotion shapes the diffusion of moralized content in social networks. *Proceedings of the National Academy of Sciences*, 114(28):7313–7318, 2017.
4. James W. Pennebaker, Ryan L. Boyd, Kayla Jordan, and Kate Blackburn. The development and psychometric properties of LIWC2015. In *Texas ScholarWorks*, 2015.
5. Pinkesh Badjatiya, Shashank Gupta, Manish Gupta, and Vasudeva Varma. Deep learning for hate speech detection in tweets. In *WWW Companion*, 2017.
6. Thomas Davidson, Dana Warmsley, Michael Macy, and Ingmar Weber. Automated hate speech detection and the problem of offensive language. In *ICWSM*, 2017.
7. Daniel Cer, Yinfei Yang, Sheng-yi Kong, Nan Hua, Nicole Limtiaco, Rhomni St John, Noah Constant, Mario Guajardo-Céspedes, Steve Yuan, Chris Tar, et al. Universal sentence encoder. *arXiv*, 1803.11175, 2018.
8. Robert Gunning. *The technique of clear writing.* McGraw-Hill, Toronto, CA, 1952.
9. Douglas Bates, Deepayan Sarkar, Maintainer Douglas Bates, and L Matrix. *lme4*, 2021. URL https://cran.r-project.org/web/packages/lme4/index.html. version 1.1.27.
10. Michael Olusegun Akinwande, Hussaini Garba Dikko, and Agboola Samson. Variance inflation factor: As a condition for the inclusion of suppressor variable(s) in regression analysis. *Open Journal of Statistics*, 5(7):754–767, 2015.
11. Mai ElSherief, Vivek Kulkarni, Dana Nguyen, William Yang Wang, and Elizabeth Belding. Hate lingo: A target-based linguistic analysis of hate speech in social media. In *ICWSM*, 2018.
12. Jason W Burton, Nicole Cruz, and Ulrike Hahn. Reconsidering evidence of moral contagion in online social networks. *Nature Human Behaviour*, 5(12):1629–1635, 2021.
13. Max Kuhn. *tidymodels*, 2022. URL https://cran.r-project.org/web/packages/tidymodels/index.html. version 1.0.0.
